# Effects of Camphor on the Teeth

**Published:** 1848-01

**Authors:** C. Spencer Bate

**Affiliations:** Surgeon Dentist.


### ARTICLE IX.

### *Effects of Camphor on the Teeth.*

**To the Editor of The London Lancet.**

SIR:—The announcement, through the medium of one or two letters in the Lancet, that camphor becomes a destructive agent when applied to the teeth, a theory so totally at variance with the long received opinion, which has induced it to become a very favorite ingredient in tooth-powders, seems, instead of instigating an inquiry into the truth of the assertion, to have caused a panic among individuals who have the habit of using the same.

That there is a disease which affects the teeth in the peculiar position referred to by Messrs. Tearne and Hunt, that has not yet been satisfactorily accounted for, must have attracted the attention of every individual who has made the diseases of teeth his study; but the assertion, that it is caused by the action of camphor on the teeth is based upon a most egregious error, since precisely the same character of disease is to be found in the mouths of individuals who never (perhaps in the course of their existence) put a brush, much less powder, inside their mouths.

Nor has the camphor, in experiments upon the teeth, the effect that has been asserted; in fact, I can discover not the least perceptible alteration upon the enamel of certain sections of teeth, which I have placed under peculiar experiments since the subject has been noticed in the Lancet. Truly, this may barely be sufficient time as a test, yet I believe that the action of the daily application of the camphor dentifrice for years to be much less than the continued action from a fortnight's immersion in the material. Then, if it is not camphor, the inquiry is suggested—What is it?

It has long been my belief, and under all cases I can (I think) make my argument hold correct, that this peculiar disease originates from an external injury inflicted upon the periosteum of the tooth, in which it originates, produced either from the continual friction of a hard tooth-brush, from the use of metal tooth-picks, or some other hard material injudiciously applied to the teeth; and also may be, and I have no doubt often is produced by the incautiousness of the use of the instruments during the operation of scaling the teeth.

If we just notice which teeth are most commonly afflicted with this disease; moreover, the peculiar position in which it occurs in teeth; I think you will see that it adds force to my opinion, while, at the same time, it equally tends to overthrow that of the camphor theory.

The teeth in which I have most commonly, in practice, noticed the disease, is first, and by far the most numerous, the canines, both upper and lower; secondly, the front upper; thirdly, the bicuspides, both upper and under, as well as the first and second molar: the front under teeth are seldom affected. In the canines, I attribute the more than equal share to liability to accident from their prominent position, and the safety of the front under teeth from the common deposit of tartar which is thrown upon them.

This disease is always upon the outer surface; for upon the palatal and lingual surfaces I do not remember ever having seen it but in one or two cases. Now if camphor were the exciting agent, it would be as liable to act upon the internal as upon the external surface, and more likely than either to act at the interstices, where a deposit of the deleterious matter might take place; but it is not so.

I am further borne out in my own theory by the observation, that in those individuals to whom I have attributed it as originating from the application of a hard brush, I have invariably found that most of the other teeth have, more or less, their fangs denuded of their natural covering, and that the time of life is past maturity; but this is not the case either when the disease takes place in youth, or in the mouths of those adults who do not make use of a tooth-brush; and in these cases it is that I attribute the disease to have originated in an injury upon the periosteum, caused by picking the teeth with pins, tooth-picks, or any hard substance.

The exact position of the tooth in which the disease originates is where the dentine of the tooth approaches nearest to the cortex, the crown being covered by an enamel which ends just where the cortex which covers the fang commences, and both gradually decreasing, unite at this point, where also the periosteum originates; and I presume it is not improbable, that in the mouths of those persons most liable to the disease, the natural cortex is peculiarly deficient.

Consequently, the disease does not progress through a deterioration of the enamel, but arises below, and eats beneath it; and I feel assured that any dentist who ever operated on a tooth will bear witness to the truth of this statement, that the enamel which is contiguous to this class of disease, is as healthy and perfect as that in the neighborhood of any other species of decay found in teeth that is friable only from want of support, caused by the diseased dentine beneath.

I am, sir, your obedient servant,

C. SPENCER BATE, *Surgeon Dentist.*

### ARTICLE X.

### *Premature Dentition and Supernumerary Teeth.*

